# mPEG-PDLLA Micelles Potentiate Docetaxel for Intraperitoneal Chemotherapy in Ovarian Cancer Peritoneal Metastasis

**DOI:** 10.3389/fphar.2022.861938

**Published:** 2022-04-06

**Authors:** Yumei Zhang, Shunli Wang, Xiaofan Duan, Xiaoxiao Xu, Yuan Gao, Jiuli Zhou, Xiaolin Xu, Jin Li

**Affiliations:** ^1^ Department of Oncology, Shanghai East Hospital, School of Medicine, Tongji University, Shanghai, China; ^2^ Department of VIP Clinic, Shanghai East Hospital, School of Medicine, Tongji University, Shanghai, China; ^3^ Department of Pathology, Tongji Hospital, School of Medicine, Tongji University, Shanghai, China

**Keywords:** ovarian cancer, peritoneal metastasis, DTX-mPEG-PDLLA micelles, intraperitoneal administration, pharmacokinetics

## Abstract

Ovarian cancer is the second most common cause of gynecological cancer death in women. It is usually diagnosed late and accompanied by peritoneal metastasis. For ovarian cancer with peritoneal metastasis, intraperitoneal (IP) chemotherapy can maintain a high drug concentration in the abdominal cavity and reduce local and systemic toxicity. Recently, docetaxel (DTX) has shown broad-spectrum antitumor activity against various malignant tumors, including ovarian cancer with peritoneal metastasis. However, DTX has limited clinical applications due to its poor water solubility, predisposition to hypersensitivity, fluid retention, and varying degrees of neurotoxicity. In this study, we prepared methoxy-poly(ethylene glycol)-block-poly(D,L-lactide) (mPEG-PDLLA) micelles loaded with DTX and developed an alternative, less toxic, more effective DTX formulation, without Tween 80, and evaluated its pharmacokinetics in the abdominal cavity and its efficacy in ovarian cancer with peritoneal metastasis. The mean diameter of DTX-mPEG-PDLLA was about 25 nm, and the pharmacokinetics of BALB/c mice *via* IP showed that the plasma exposure of DTX-mPEG-PDLLA was about four times lower than that of DTX. Importantly, DTX-mPEG-PDLLA was significantly more effective than DTX and prolonged the survival period in a SKOV-3 ovarian cancer peritoneal metastasis model. Moreover, the apoptosis rate was significantly increased *in vitro*. Based on these findings, it is expected that DTX-mPEG-PDLLA can enhance efficacy against ovarian cancer peritoneal metastasis, while reducing toxic side effects, and has the potential to be used in the clinical treatment of peritoneal metastatic cancer.

## Introduction

Ovarian cancer is the second most common gynecological malignancy. It is estimated that there were approximately 295,414 new cases and 184,799 deaths from ovarian cancer in 2018 (Bray et al., 2018). The diagnosis of ovarian cancer is mostly at an advanced stage of the disease, often with peritoneal metastasis, with a poor prognosis (Kuroki and Guntupalli, 2020; Menon et al., 2021). Generally, compared with intravenous administration, intraperitoneal (IP) chemotherapy is expected to provide a higher drug concentration and lower systemic toxicity in the peritoneal cavity (de Bree et al., 2006; Menon et al., 2021). Moreover, it has been shown that IP chemotherapy was associated with prolonged survival of patients with peritoneal metastases from ovarian cancer (Elit et al., 2007; Shi et al., 2019).

Docetaxel (DTX), a member of the taxane family, has become one of the most effective antitumor drugs against various malignant solid tumors such as ovarian cancer with peritoneal metastasis. DTX can induce polymerization of tubulin monomers and inhibit their depolymerization, leading to mitotic arrest in the G2/M phase of the cell cycle (Montero et al., 2005; Wang et al., 2014; Yamada et al., 2019). Furthermore, studies have confirmed that the concentration of DTX administered *via* IP was 100-fold higher and maintained for a longer time than that from intravenous administration (Shimada et al., 2010). However, DTX has limited clinical applications due to its poor water solubility, prone to hypersensitivity, fluid retention, and varying degrees of neurotoxicity, and because Tween 80 and 95% ethanol are needed for DTX preparations in the clinical formulation (Gong et al., 2020). In view of these drawbacks, efforts have been focused on changing the formulation and developing alternative, less toxic, and more effective DTX preparations (Lee et al., 2011; Fan et al., 2014; Saw et al., 2017; Ghassami et al., 2018; Liu et al., 2019; Kim et al., 2020).

In recent years, as a superior drug delivery system, self-assembled polymer micelles with amphiphilic blocks represent a class of nanocarriers with a well-defined core-shell structure, which can overcome the poor water solubility of antitumor drugs, and enhance their delivery to tumor sites through passive or active targeting, thereby reducing adverse effects to healthy tissues (Lu et al., 2018; Jo et al., 2021; Xu et al., 2021; Yang et al., 2021; Zheng et al., 2021). In addition, the bioavailability of drugs at the tumor site has been increased through an enhanced permeability and retention (EPR) mechanism (Hu et al., 2020; Tan LW et al., 2017; Younis et al., 2022). Therefore, as one of the most effective chemotherapy drugs for ovarian cancer, DTX-loaded micelles may enhance local or systemic antitumor effects.

**FIGURE 1:**
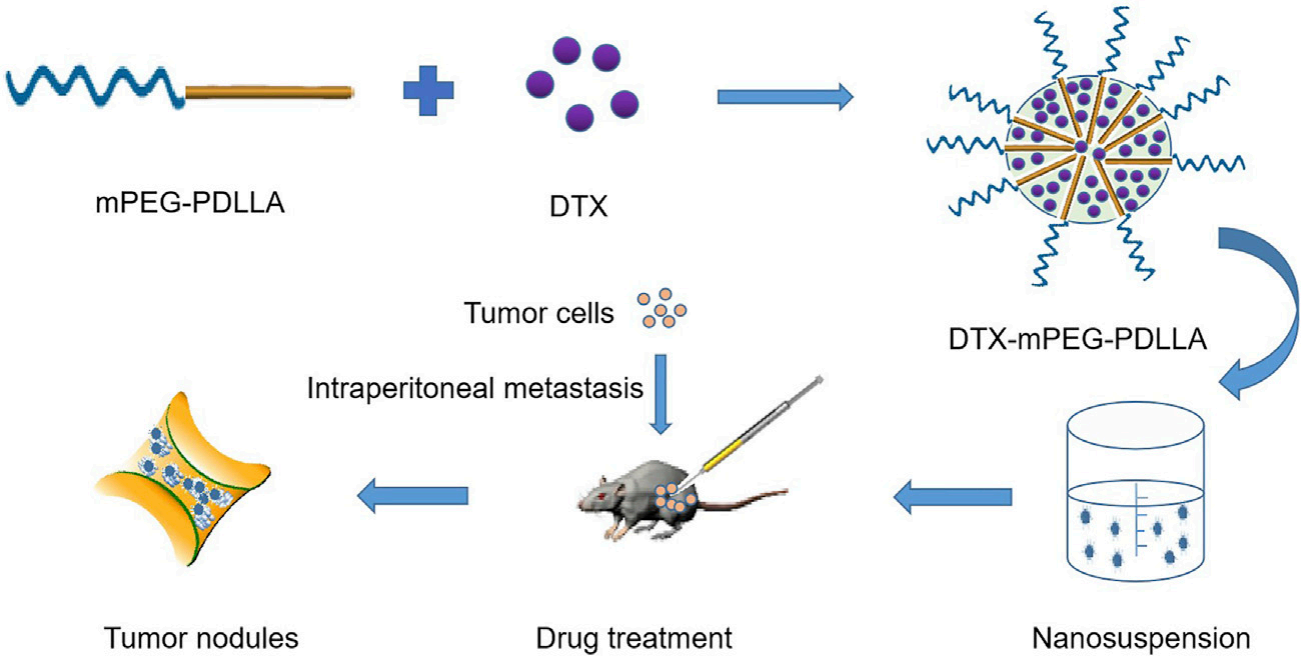
Schematic of the synthesis and intraperitoneal administration of DTX-mPEG-PDLLA micelles. The DTX-mPEG-PDLLA micelles were prepared with DTX and mPEG-PDLLA polymers by a self-assembly method. The drugs were delivered by the micelles after IP administration for an antitumor effect *in vivo*.

In this work, we reported the development of a DTX micelle using a non-toxic and biodegradable amphiphilic diblock copolymer, mPEG-PDLLA, which has improved the poor aqueous solubility and substantial toxic side effects of DTX, and has been approved by the Food and Drug Administration (FDA) (Hao et al., 2017; He et al., 2020). Furthermore, we used the marketed formulation of DTX as a control treatment to evaluate the efficacy of DTX-mPEG-PDLLA micelles administered *via* IP in a SKOV-3 ovarian cancer peritoneal metastasis model. The characterization of DTX-mPEG-PDLLA micelles was verified, and the pharmacokinetics and antitumor efficacy in peritoneal metastasis of ovarian cancer after IP administration were compared with free DTX (Figure 1).

## Meterials and Methods

### Cell Lines and Animals

The SKOV-3 human ovarian adenocarcinomal cell line (Cell Bank of Chinese Academy of Sciences, Shanghai, China) was cultured in Roswell Park Memorial Institute 1640 medium (RPMI 1640, Gibco Inc., Billings, MT, United States) containing 10% fetal bovine serum (FBS, Gibco Inc.) with 100 U/ml penicillin and 100 mg/ml streptomycin in a 37°C incubator (Thermo Fisher Scientific, Waltham, MA, United States) supplied with 5% carbon dioxide (CO_2_). DTX was purchased from Jiangsu Aosaikang Pharmaceutical Co., Ltd. (Nanjing, China). Cell Counting Kit-8 (CCK-8) was obtained from Dojindo Laboratories (Kumamoto, Japan).

Female BALB/c nude mice and male ICR mice (4–6 weeks old) were used for *in vivo* antitumor experiments and pharmacokinetic studies. The animals were purchased from the Laboratory Animal Center of jiesijie (Shanghai, China) and were separately housed in groups of five at a controlled temperature of 24–26°C, with a 12 h alternate day and night/light and dark. All mice were provided standard laboratory food and free-to-drink autoclaved tap water. All animal procedures were performed in accordance with the protocols approved by the Institutional Animal Ethics Committee of Tongji University (Shanghai, China). During the entire experiment, all mice were treated humanely. Endpoints requiring euthanasia included inactivity, weight loss of more than 20%, excessive muscle atrophy, and hypothermia.

### Critical Micelle Concentration

The pyrene fluorescence method was used to determine the CMC of the copolymers. Before fluorescence detection, the sample was prepared according to the instructions, the emission wavelength was set to 330 nm, and the excitation spectrum scanning range was 300–400 nm. The fluorescence intensity ratio was calculated and plotted at 333 and 339 nm (I_333_/I_339_). The intersection of two linear regressions was considered the CMC value.

### Preparation of DTX-mPEG-PDLLA Micelles

The thin-film hydration method was used to prepare DTX-mPEG-PDLLA micelles. Briefly, DTX (20 mg) and mPEG-PDLLA (480 mg) were co-dissolved in 1 ml of methanol. Next, mPEG-PDLLA and DTX stock solution were mixed and heated over 47°C rotary steam to remove the solvent. Subsequently, 40 μl of normal saline was added and hydrated at 60°C to prepare the DTX-mPEG-PDLLA micelles.

### Characterization of DTX-mPEG-PDLLA Micelles

To detect drug loading (DL) and encapsulation efficiency (EE), 20 mg of lyophilized DTX-mPEG-PDLLA was dissolved in acetonitrile. The sample was determined by high performance liquid chromatography (HPLC, Shimadzu LC-20AT, Japan). The values of DL and EE of DTX-mPEG-PDLLA were calculated by the equations: DL (%) = (weight of DTX in micelles/weight of DTX-mPEG-PDLLA) ×100%; EE (%) = (weight of DTX in micelles/weight of DTX added) ×100%.

Next, the particle size and zeta potential of DTX-mPEG-PDLLA were measured by Nano-ZS 90 Nanosizer (Zetasizer Nano; Malvern, Panalytical, United Kingdom) after dilution with distilled water. Some DTX-mPEG-PDLLA micelle suspension was dropped on the special copper net for the transmission electron microscope (TEM; Tecnai G2 Spirit BioTWIN, Huck Institutes of Life Sciences, University Park, PA, United States) and allowed to dry naturally. The morphology of DTX-mPEG-PDLLA was observed after the sample was prepared.

### *In Vitro* Drug Release Behavior

Dialysis was used to evaluate the *in vitro* release of DTX-mPEG-PDLLA micelles. During the experiment, 1 ml DTX-mPEG-PDLLA micelles containing 20 mg DTX were placed in a dialysis bag, and 0.5% Tween 80 containing 0.01 mmol/L phosphate buffer (pH 7.4) was used as the release medium. During this process, 1 ml of the sample was taken at different time intervals (0, 0.5, 1, 2, 4, 6, 8, and 12 h), and blank release medium was added. The released DTX was quantified by the HPLC method.

### Cell Uptake Assay

Fluorescence microscopy was used to observe the cellular uptake. In this study, coumarin-6 (C6) as a fluorescent probe was loaded into the micelles (C6-mPEG-PDLLA). The concerntration of C6 and mPEG-PDLLA for the preparation was 50 μg/ml and 1 mg/ml, respectively. SKOV-3 cells were seeded in six-well plates at a density of 1×10^6^ cells/well and cultured for 24 h. C6-mPEG-PDLLA micelles were diluted to 2.5 μg/ml, then C6 and C6-mPEG-PDLLA micelles were added to the six-well plates, and incubated for 2 h at 37°C in the dark. Cells were washed with phosphate buffer saline (PBS), fixed with 4%-paraformaldehyde, and dyed with 4ʹ,6-diamidino-2-phenylindole (DAPI) for nuclear staining. Then, cell uptake of SKOV-3 was observed and scanned by fluorescence microscopy (Leica DMi8; Leica Microsystems Ltd., Wetzlar, Germany).

### Cell Viability Assay

The CCK-8 detection method measured cell viability and was used in accordance with the manufacturer’s instructions. SKOV-3 cells were seeded in 96-well plates at a density of 1×10^4^ cells/well, and treated as expected after 24 h of culture. Reagent (10 µl) was added to the wells according to the experimental design, and the plates were incubated at 37°C for 2 h. An optical density of 490 nm was achieved on an enzyme-labeled instrument (Infinite M200 pro; Tecan, Switzerland).

### Cell Cycle and Cell Apoptosis Analysis

The cell cycle and apoptosis detection kit (Beyotime; Shanghai, China) were used in accordance with the manufacturer’s instructions. SKOV-3 cells were seeded in a 24-well plate at a density of 1×10^5^ cells/well for 24 h, and then treated with a control, mPEG-PDLLA, DTX, or DTX-mPEG-PDLLA at 37°C for 48 h. Finally, the cells were analyzed using the CytExpert flow cytometer (Beckman Coulter, Brea, CA, United States).

### Pharmacokinetics Study

The male ICR mice were divided into the DTX and DTX-mPEG-PDLLA micelle groups. DTX was dissolved using commercially exclusive solvent and DTX-mPEG-PDLLA micelles were dissolved in normal saline (NS). After the mice were treated with IP administration of DTX or DTX-mPEG-PDLLA micelles 10 mg/kg, the samples were collected from orbital blood at different time points (5 mice at each time point). Blood samples were collected at 0, 0.033, 0.083, 0.167, 0.25, 0.5, 1, 2, 3, 6, 8, and 24 h. The plasma was separated and extracted with acetonitrile solution containing 0.1% formic acid, and the samples were blown with nitrogen at a temperature of 40°C until concentrated. The dry residues were dissolved in water with 0.1% formic acid for liquid chromatograph-mass spectrometer/mass spectrometer (LC-MS/MS) analysis. WinNonlin software (Certara, Princeton, NJ, United States) was used for data analysis.

### *In Vivo* Tumor Model

To evaluate the therapeutic effect of DTX-mPEG-PDLLA in the ovarian cancer peritoneal metastasis model, female BALB/c nude mice (4–6 weeks old) were reared with five mice in each group. Mice were inoculated intraperitoneally with 1×10^8^ SKOV-3 cells. After measuring the tumor diameter at the IP inoculation site to about 1 mm, the tumor-bearing mice were randomly divided into seven groups and received NS, DTX (10, 20, and 40 mg/kg), and DTX-mPEG-PDLLA micelles (10, 20, and 40 mg/kg) were administered to the abdominal cavity *via* IP, once every 7 days for a total of two administrations and weighed every 3 days during the period. On the 24th day after the administration, all the mice were killed by cervical dislocation, and the tumor nodules were immediately harvested and weighed. Similarly, to detect drug distribution in tumors and tissues, tumors and some organs of mice were harvested after being killed by IP administration for 1 h at a drug dose of 10 mg/kg for further analysis. The concentration of DTX was analyzed by HPLC.

### Statistical Methods

All data were expressed as mean ± standard deviation (SD). Statistical analysis was performed using GraphPad Prism 6 software (San Diego, CA, United States). The Student’s *t*-test or one-way analyses of variance (ANOVA) were used to compare the differences in groups. A confidence level of 95% was used for all analyses (*α*-value = 0.05). Results were summarized as ****p < 0.0001, ***p < 0.001, **p < 0.01, and *p < 0.05.

## Results

### Preparation and Characterization of DTX-mPEG-PDLLA Micelles

**FIGURE 2:**
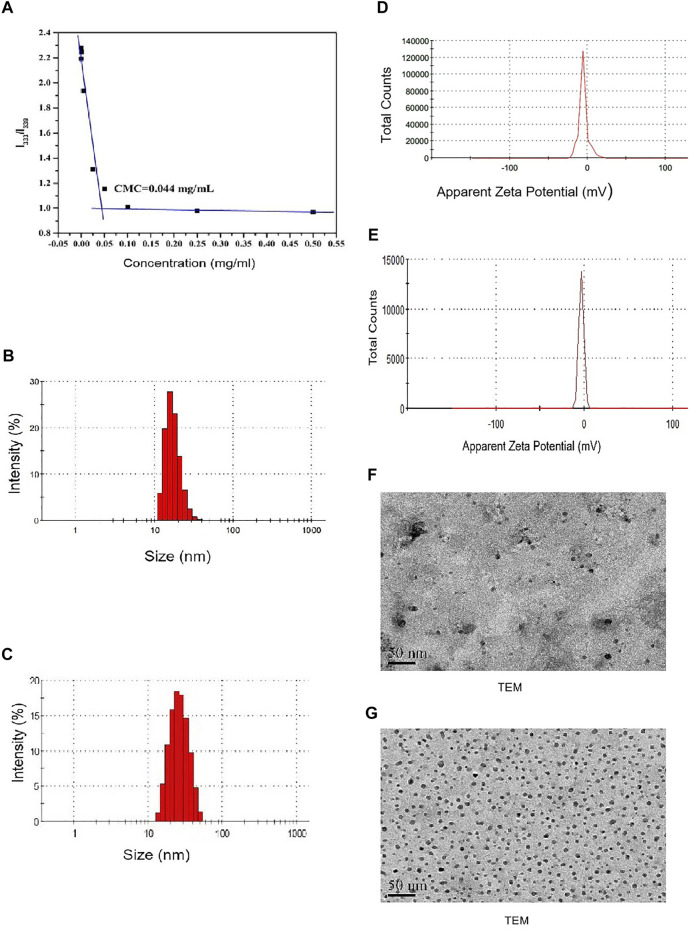
Characterization of DTX-mPEG-PDLLA micelles. **(A)** CMC measurement of mPEG-PDLLA by a pyrene fluorescence probe. **(B,C)** Particle size distribution of mPEG-PDLLA and DTX-mPEG-PDLLA micelles. **(D,E)** Zeta potential of mPEG-PDLLA and DTX-mPEG-PDLLA micelles. **(F,G)** TEM images of mPEG-PDLLA and DTX-mPEG-PDLLA micelles.

In this work, the molecular weight of the mPEG-PDLLA polymer was about 4,000 and of mPEG and PDLLA were each 2000. The CMC value of DTX-mPEG-PDLLA was 0.044 mg/ml (Figure 2A). Such a low CMC value ensured that the copolymers self-assembled into stable micellar carriers, as expected, which was essential for effective delivery of antitumor drugs to the tumor sites.

DTX-mPEG-PDLLA micelles were prepared by the thin-film hydration method. In the process of evaporating and removing alcohol, DTX and copolymers were distributed as an amorphous substance. After water was added, mPEG-PDLLA copolymers self-assembled into core–shell structured micelles with DTX encapsulated in the core.

The DL and EE of DTX-MPEG-PDLLA micelles were 4.27 ± 0.36% and 99.24 ± 0.62%, respectively, which indicated that mPEG-PDLLA had high encapsulation efficiency as a micelle material. Meanwhile, the mean particle size of the mPEG-PDLLA and DTX-mPEG-PDLLA micelles were about 23 and 25 nm, respectively (Figures 2B,C). Zeta potential of the mPEG-PDLLA and DTX-mPEG-PDLLA micelles were about −5.02 mV and −3.13 mV, respectively (Figures 2D,E). Importantly, all were close to each other. In addition, according to the narrow particle size distribution of DTX micelles, the polydispersity index (PDI) was less than 0.12, indicating that there was good monodispersity. Combined with the results of the TEM micrograph (Figures 2F,G), both the mPEG-PDLLA and DTX-mPEG-PDLLA micelles had a regular shape, which was consistent with the analysis of the particle size.

**FIGURE 3:**
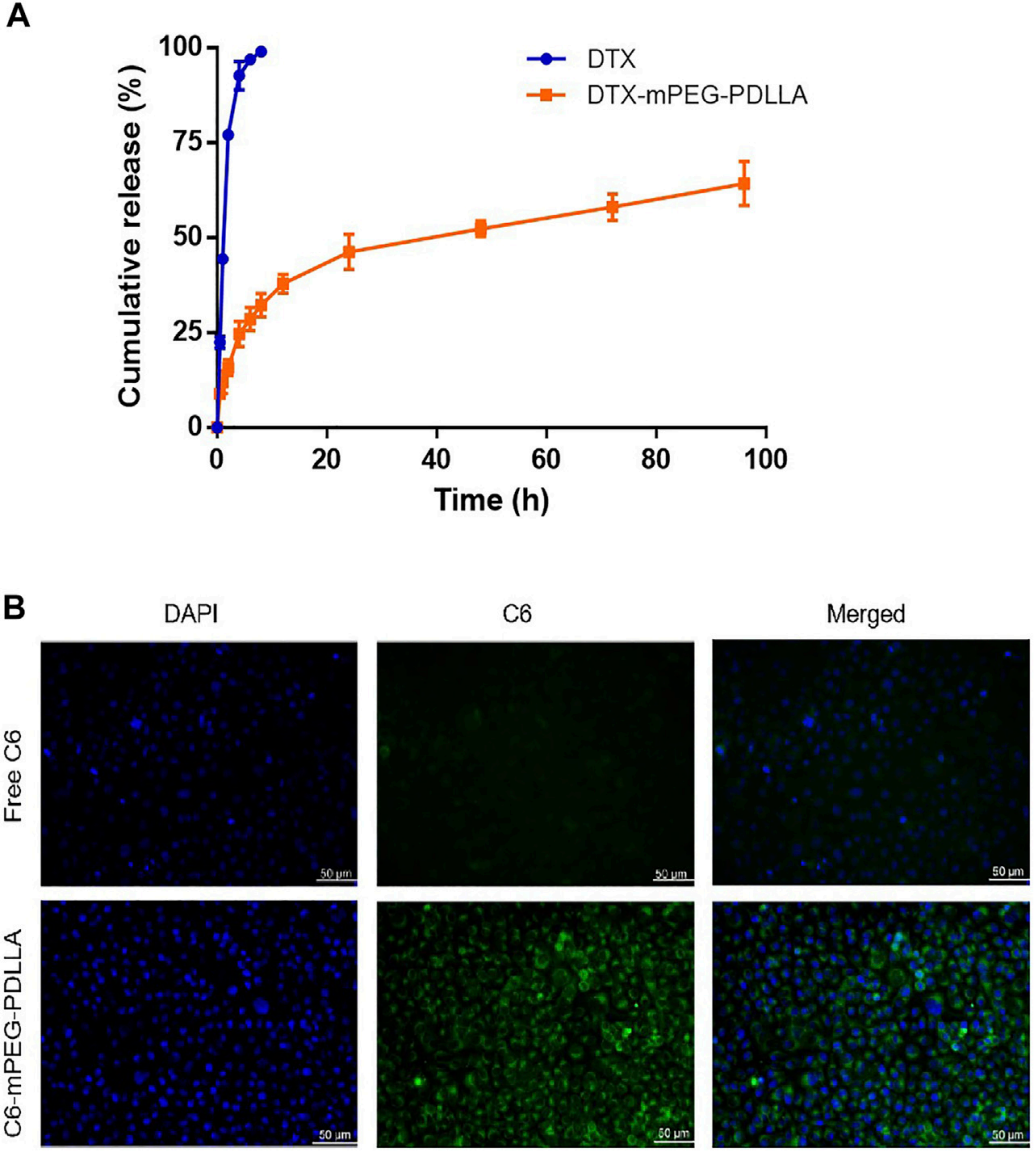
DTX release from DTX-mPEG-PDLLA micelles *in vitro* and cellular uptake of C6-mPEG-PDLLA micelles. **(A)** Release characteristics of DTX and DTX-mPEG-PDLLA micelles *in vitro*. The data were analyzed as mean ± SD (*n* = 3). **(B)** Cellular uptake in SKOV-3 cells.

The dialysis method was used to investigate whether DTX-mPEG-PDLLA micelles delayed the release time of DTX. Approximately, 45% of DTX was released rapidly in the first hour of DTX injection, and DTX was almost released completely within 6 h, with a cumulative release amount close to 96%. In contrast, less than 30% of DTX-mPEG-PDLLA micelles were released in 6 h and 58% in 72 h (Figure 3A). These findings showed that DTX-mPEG-PDLLA micelles had a significantly slower release (*p* < 0.05). Furthermore, cellular uptake of C6-mPEG-PDLLA was performed in SKOV-3 cells. DAPI was used to stain the nucleus for blue fluorescence, and green fluorescence represented the release of C6 in SKOV-3 cells. After incubation for 2 h, the green fluorescence in the C6-mPEG-PDLLA group was stronger than that in the free C6 group (Figure 3B).

### Performance of DTX-mPEG-PDLLA in Ovarian Cancer Cells

**FIGURE 4:**
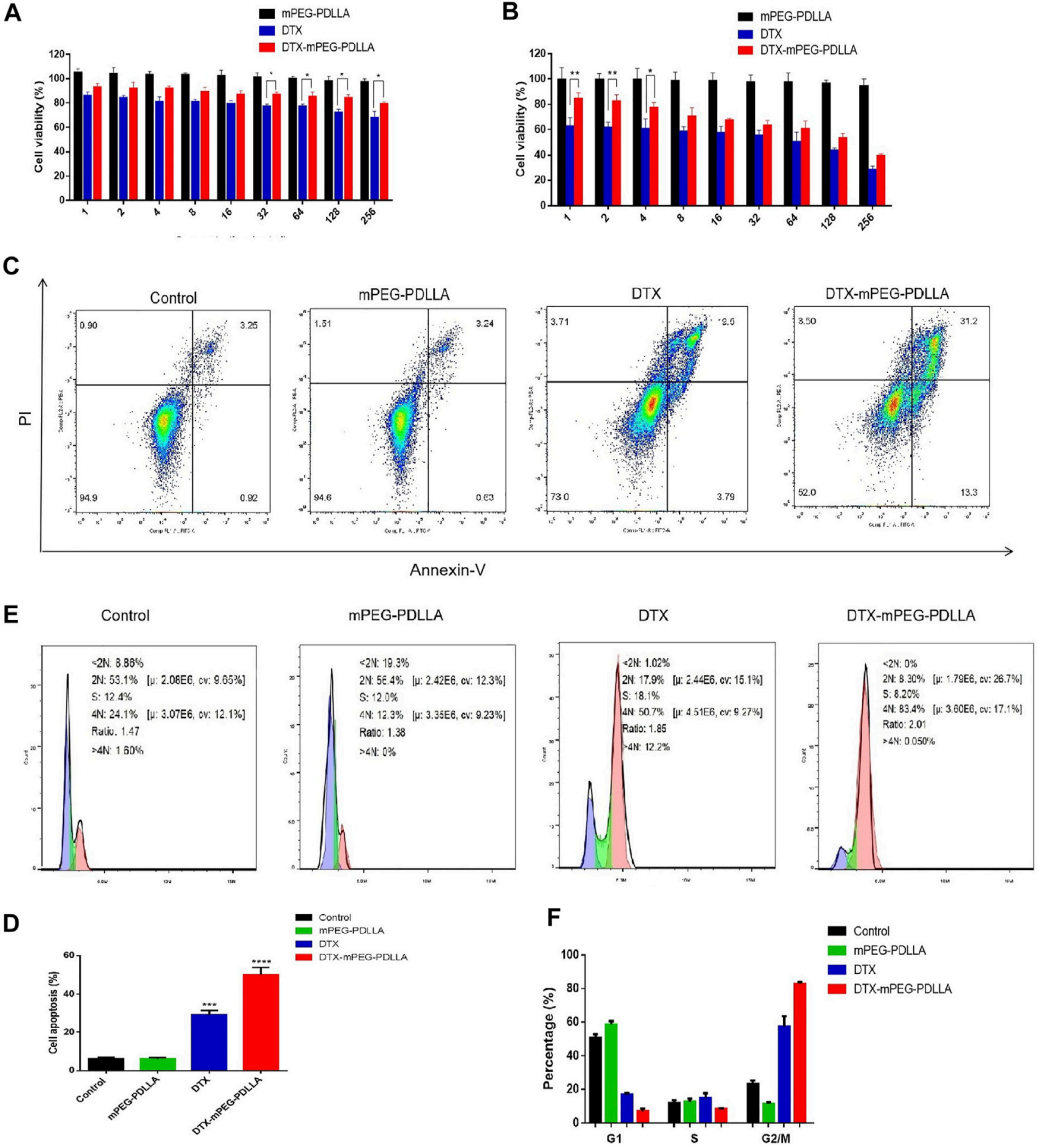
Antitumor activity of DTX-mPEG-PDLLA micelles in ovarian cancer cells. **(A,B)** Cytotoxicity of mPEG-PDLLA, DTX, and DTX-mPEG-PDLLA micelles in SKOV-3 human ovarian cancer cells treated for 24 and 48 h, respectively (*n* = 3, mean ± SD); *p < 0.05 and **p < 0.01. **(C)** Apoptosis induction of SKOV-3 cells treated with mPEG-PDLLA, DTX, and DTX-mPEG-PDLLA for 48 h analyzed by the Annexin V-FITC Apoptosis Detection Kit. **(D)** Results representing three-independent experiments; ***p < 0.001, ****p < 0.0001 vs. the control group. **(E,F)** Cell cycle assays and analysis of mPEG-PDLLA, DTX, and DTX-mPEG-PDLLA micelles in SKOV-3 cells treated for 48 h, respectively (*n* = 3, mean ± SD).

*In vitro* CCK-8 was used to evaluate the cytotoxicity of DTX-mPEG-PDLLA and DTX against SKOV-3 ovarian cancer cells. The results showed that both DTX-mPEG-PDLLA and DTX effectively inhibited the proliferation of SKOV-3 cells in a dose-dependent manner, and the blank micelles had no cytotoxicity. Moreover, the cell viability of the DTX-mPEG-PDLLA group was lower than that of the DTX group after 24 and 48 h of treatment (Figures 4A,B), which may be related to the delayed release of micelles. In short, there was no significant difference between the two groups.

Next, to further explore the antiproliferative effect of DTX-mPEG-PDLLA micelles in ovarian cancer cells, we examined apoptosis of SKOV-3 cells after 48 h of different treatments. First, the apoptosis rates of the two drug groups were significantly higher than that of the control group and the blank mPEG-PDLLA group. Second, compared with the DTX group, the apoptosis rate of the DTX-mPEG-PDLLA group increased significantly, while the mPEG-PDLLA group did not increase compared with the control group, which was consistent with the cell viability test (Figures 4C,D).

It is known that DTX is a specific drug acting on the M-phase in the cell cycle, which promotes the aggregation of tubules into stable microtubules and inhibits their aggregation, resulting in the formation of stable non-functional microtubule bundles, thus destroying the mitosis of tumor cells (Liang et al., 2021). Consequently, we performed an analysis of the cell cycle of the two drugs. The results showed that both DTX and DTX-mPEG-PDLLA prominently resulted in mitosis inhibition and G2/M arrest, with mean values of 60 and 84% in SKOV-3 cells, respectively (Figures 4E,F). Similarly, apoptosis induction and cell cycle arrest of blank mPEG-PDLLA micelles were no different from those of the control group, which showed that they had good cytocompatibility.

### Plasma Pharmacokinetics

**FIGURE 5:**
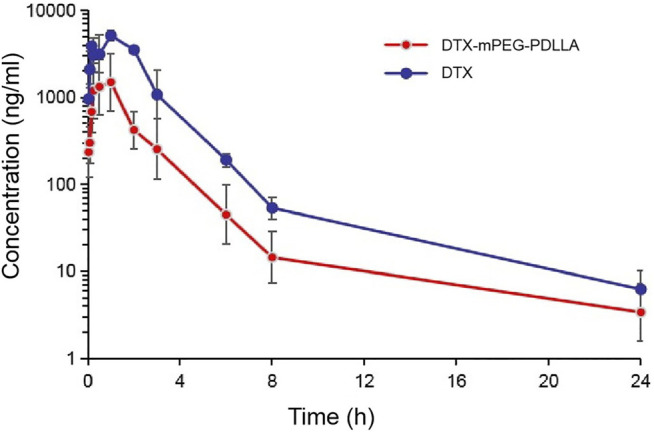
Pharmacokinetics of DTX-mPEG-PDLLA micelles by IP administration *in vivo*. Plasma concentration–time curves of free DTX and DTX-mPEG-PDLLA micelles in mice *via* IP administration (*n* = 6).

*In vivo* IP pharmacokinetics were investigated in ICR mice, and the plasma concentration–time curve of the two drugs after IP administration is shown in Figure 5. The main pharmacokinetic parameters are presented in Table 1. The area under the curve (AUC) from time zero to the last measurable time (AUC_last_; 12.77 ± 0.16 h*µg/ml) and AUC from time of dosing extrapolated to infinity, based on the last observed concentration (AUC_INF-obs_; 12.80 ± 0.13 h*µg/ml) for DTX were significantly higher than that for DTX-mPEG-PDLLA (3.10 ± 0.06 and 3.13 ± 0.04 h*µg/ml), respectively. The values of maximum serum concentration (C_max_) also showed a significant increment in DTX (5.16 ± 0.03 μg/ml) compared to DTX-mPEG-PDLLA (1.49 ± 0.04 μg/ml), and the half-life of DTX (4.11 ± 0.01 h) was lower than that of DTX-mPEG-PDLLA (5.62 ± 0.02 h). Therefore, compared with the DTX group, a lower concentration of DTX-mPEG-PDLLA was observed in plasma, and the mean values of AUC_last_ and C_max_ were less by about four times. This suggests that there is a significant difference in the amount of exposure of the two drugs in plasma by IP administration. This may be affected by the following two factors. On one hand, DTX was stabilized and delayed release from the DTX-mPEG-PDLLA micelles into peritoneal cavity, while DTX was released rapidly in the free DTX group. On the other hand, capillary density was relatively low in the peritoneum. Generally, the growth of tumor cells is often accompanied by angiogenesis, and the capillaries of tumor tissue have a porous structure, which is less complete than normal tissue. As a result, lipid particles and macromolecules such as DTX-mPEG-PDLLA micelles could pass through the leaky capillaries by the EPR effect, resulting in retention of these molecules in tumor tissue and slower penetration into the blood circulation than DTX (Van Der Speeten et al., 2009; Shi et al., 2017; Zheng et al., 2021).

**TABLE 1:** Pharmacokinetic parameters of the DTX and DTX-mPEG-PDLLA micelles after IP administration in mice.

Parameter DTX DTX-mPEG-PDLLA		
AUC_last_ (h * µg/ml)	12.77 ± 0.16	3.10 ± 0.06
AUC_INF-obs_ (h * µg/ml)	12.80 ± 0.13	3.13 ± 0.04
C_max_ (µg/ml)	5.16 ± 0.03	1.49 ± 0.04
MRT last (h)	2.05 ± 0.02	1.99 ± 0.01
T_1/2_ (h)	4.11 ± 0.01	5.62 ± 0.02

### Antitumor Efficacy of DTX-mPEG-PDLLA Micelles *via* IP Administration *In Vivo*

**FIGURE 6:**
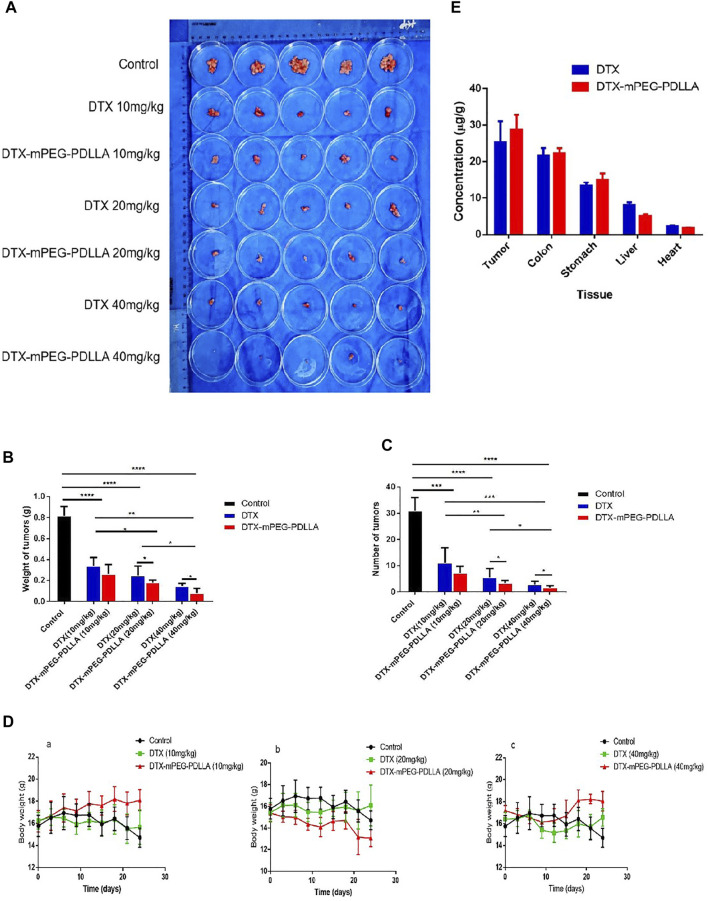
Antitumor effect and tissue distribution of DTX-mPEG-PDLLA micelles by IP administration in ovarian cancer peritoneal metastasis. **(A)** Photographs of peritoneal cavity tumors in each group on day 24 after IP administration. **(B)** Analysis of tumor nodules weight. **(C)** Numbers of tumor nodules. **(D)** Changes in body weight of mice in the 10 mg/kg (a), 20 mg/kg groups (b), 40 mg/kg groups (c), and the control group, which was injected with NS by IP administration. **(E)** Comparison of DTX and DTX-mPEG-PDLLA in tissues and tumors 1 h by IP administration at 10 mg/kg dosage. *p < 0.05, **p < 0.01, ***p < 0.001, and ****p < 0.0001.

The antitumor efficacy of DTX-mPEG-PDLLA micelles *via* IP administration were evaluated in a SKOV-3 ovarian cancer peritoneal metastasis model. Thirty-five BALB/c nude mice were divided into seven groups treated with NS, DTX (10, 20, 40 mg/kg), or DTX-mPEG-PDLLA (10, 20, and 40 mg/kg). At each corresponding dose injected, all of the DTX-mPEG-PDLLA micelles and DTX showed significant tumor suppression, and the numbers of tumor nodules were decreased compared with the control group (Figure 6A, *p <* 0*.*001). Meanwhile, combined with the weight and size of the tumor nodules, it was observed that the high-dose group (40 mg/kg, *p <* 0*.*01) had significantly better antitumor effects than the middle and low-dose groups (20 and 10 mg/kg, respectively). Moreover, the growth of peritoneal cavity tumors was inhibited more significantly in the DTX-mPEG-PDLLA group than in the DTX group (Figures 6B,C and *p <* 0*.*05). During the study, the weight of each mouse was measured and recorded every 3 days, and the corresponding curves are shown in Figure 6D. In the negative control groups, in which NS was administered to the animals, the body weight changes showed a trend to increase first and then decrease after 12 days as the tumors were left untreated. In the DTX-treated groups, the body weight of these mice showed relatively unchanged and no statistical difference in the 10 and 20 mg/kg groups, but an upward trend after day 12 in the 40 mg/kg group, due to tumor inhibition by DTX. Among the animals that were treated with DTX-mPEG-PDLLA micelles, the body weight of the 40 mg/kg group fluctuated throughout the study; however, the average weight of the mice trended upward after the 9th day, which may indicate the reduced toxicity of DTX-mPEG-PDLLA micelles in combination with a more durable tumor suppressive effect. In addition, the drug distribution in tissues is shown in Figure 6E, and the concentration of DTX was measured 1 h after IP administration at a dose of 10 mg/kg. Compared with the DTX group, the concentration of DTX in the tumors in the DTX-mPEG-PDLLA group was higher than that in the free DTX group, and the absorption was more. The result suggested that the anti-tumor efficacy was improved due to the EPR effect. Moreover, the concentration in the DTX-mPEG-PDLLA group was higher in the colon and stomach than in the liver and lowest in the heart, reducing systemic toxicity of DTX, although no significant difference was shown at 10 mg/kg dosage.

**FIGURE 7:**
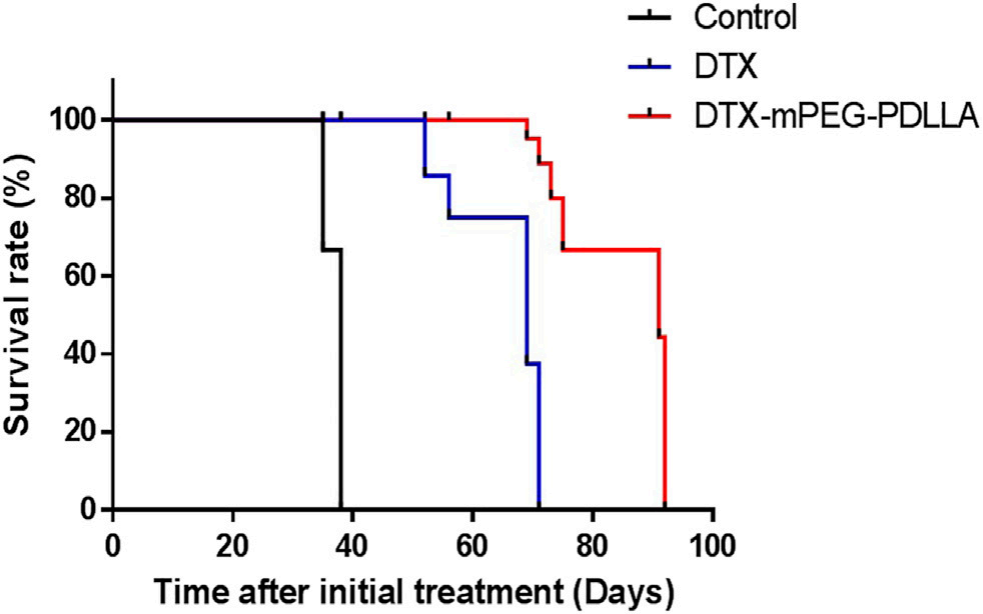
Survival analysis of DTX-mPEG-PDLLA micelles *via* IP administration in ovarian cancer peritoneal metastasis. Survival curves for SKOV-3 tumor-bearing nude mice receiving different treatments. Mice were treated with NS, DTX, or DTX-mPEG-PDLLA micelles on days 1 and 7.

Furthermore, the survival of mice injected with DTX and DTX-mPEG-PDLLA at a dose of 40 mg/kg by IP administration was examined, and NS was injected as a control. In this analysis, a SKOV-3 ovarian cancer peritoneal metastasis model was established, and the mice were divided into three groups with six mice in each group. After all the mice were subjected to IP administration according to the experimental design, the mice in the control group died within 40 days with a median survival time of 38 days, which was related to the rapid growth of the tumors. The median survival in the DTX and DTX-mPEG-PDLLA groups was 69 and 91 days, respectively (Figure 7). The results indicate that the overall survival of DTX-mPEG-PDLLA micelles was prolonged significantly compared with the DTX and control groups.

## Discussion

Most ovarian cancer cases are diagnosed at an advanced stage with peritoneal or upper abdominal organ metastasis, and the 5 y survival rate is less than 48% (van Baal et al., 2018; Kuroki and Guntupalli, 2020; Mogi et al., 2021). Currently, many studies have shown that delivering antitumor drugs to the peritoneal cavity *via* IP administration has a survival advantage over intravenous chemotherapy (Armstrong et al., 2006; Gupta et al., 2019; Shi et al., 2019; Kuroki and Guntupalli, 2020). However, there are several barriers to IP administration, including higher toxicity rates and worse quality of life, including gastrointestinal adverse events, neutropenia, and neurotoxicity (Kuroki and Guntupalli, 2020; Liu et al., 2021).

Currently, drugs such as DTX, which is one of the most effective drugs for ovarian cancer treatment, have limited IP administration due to their severe side effects (de Bree et al., 2006; Gong et al., 2020). It is well known that DTX has poor water solubility, high toxicity, and low bioavailability, even though its IP penetration is far greater than that for paclitaxel and cisplatin *via* IP administration (Shimada et al., 2010; Kuroki and Guntupalli 2020). However, with the advent of biocompatibility and biodegradable polymers, the development of suitable polymer micellar drug delivery systems coupled with sustained drug release has made excellent carriers for antitumor drugs (Gao et al., 2015; Tan L et al., 2017; Li et al., 2020; Lu et al., 2020; Chen et al., 2021). Moreover, self-assembly and passive targeting of EPR might contribute to a greater therapeutic effect (Tan L et al., 2017; Lu et al., 2020; Li et al., 2020; Giram et al., 2021).

In this work, we have developed mPEG-PDLLA as a micellar carrier for sustained and controlled release delivery of DTX, which has overcome the initial disadvantages by reducing toxicity, improving solubility, and enhancing targeting to tumor tissue. Furthermore, a therapeutic effect of DTX-mPEG-PDLLA on SKOV-3 ovarian cancer with peritoneal metastasis was detected.

First, the low CMC value ensured the stability of DTX-mPEG-PDLLA micelles when diluted *in vivo*, which was essential for drug delivery. Meanwhile, DTX-mPEG-PDLLA micelles were confirmed to have a high percentage for DL and EE. The average diameter and zeta potential of DTX-loaded micelles were about 25 nm and −3.13 mv, respectively. Combined with morphological examination, the results demonstrated that DTX-loaded micelles had uniform particle size, stable drug loading, and sustained drug release behavior. Compared with DTX injection, DTX-mPEG-PDLLA micelles had slower release, which may be attributed to the molecular structural and preparation characteristics of polymeric micelles. C6-mPEG-PDLLA with a stronger green fluorescence than the free C6 group was observed in SKOV-3 cells, suggesting that micellar polymers had better uptake in ovarian cancer cells and produced EPR effects. These characteristics imply that the application of DTX-mPEG-PDLLA micelles in drug carriers was feasible and successful, reducing the exposure of DTX to healthy tissues while increasing the accumulation of concentration in tumor tissues.

In*in vitro* experiments, CCK-8 results indicated that DTX-mPEG-PDLLA micelles had comparable cytotoxicity to DTX. Furthermore, apoptosis analysis showed that, compared with DTX, the percentage of apoptotic SKOV-3 cells in DTX-mPEG-PDLLA micelles increased significantly after 48 h of treatment. Additionally, analysis of the cell cycle demonstrated that the G2/M phase of SKOV-3 cells was prominently arrested after DTX-mPEG-PDLLA micelles and DTX given for 48 h compared with the control group, with a more significant effect for DTX-mPEG-PDLLA micelles than that for the DTX group. These findings indicated that micellar preparations enhanced the cell cycle effects of DTX.

It has been reported that most of the patients are diagnosed with ovarian cancer with abdominal cavity tumor nodules or ascites (van Baal et al., 2018; Yousefi et al., 2020), and previous studies have shown that IP infusion chemotherapy has many advantages in the treatment of ovarian cancer with peritoneal metastasis, including minimal invasion, higher drug concentration in the local area, and lower systemic toxicity (de Bree et al., 2006; Zahedi et al., 2012; Kwa and Muggia, 2014; Shi et al., 2019).

In this study, the pharmacokinetics showed that there was a significant difference in plasma exposure in ICR mice after IP administration, which was markedly elevated in DTX-treated mice compared with DTX-mPEG-PDLLA treated mice. The peak mean plasma level in the DTX group was approximately fourfold higher than that in the DTX-mPEG-PDLLA group. These findings are consistent with the characteristics of DTX-loaded mPEG-PDLLA micelles, such as slow and controlled release, and long circulation.

In line with the pharmacokinetics, the results of DTX-mPEG-PDLLA micelles *via* IP administration showed a significant anticancer effect on the nude mice bearing SKOV-3 ovarian cancer with peritoneal metastasis. It was also considered that the increased survival of BALB/c nude mice treated with DTX-mPEG-PDLLA micelles was probably due to the better antitumor efficacy of mPEG-PDLLA micelles as a delivery system for DTX. Furthermore, both mPEG and PDLLA have been approved by the FDA, and have good biocompatibility and degradable absorption (Hao et al., 2017; Byagari et al., 2014). They are suitable for antitumor drug delivery systems. Moreover, mPEG-PDLLA maintained the sustained release mode of DTX over time, thereby exerting a better antitumor effect in SKOV-3 ovarian cancer with peritoneal metastasis over DTX when IP administered.

## Conclusion

In summary, we investigated the characterization of DTX-mPEG-PDLLA micelles and the inhibition of proliferation of ovarian cancer cells, as well as the plasma pharmacokinetics after IP administration and the antitumor efficacy in an ovarian cancer peritoneal metastasis model. The results have demonstrated that DTX-mPEG-PDLLA micelles have advantages for IP perfusion therapy, including prominent inhibition of abdominal tumor growth, sustained local or systemic drug exposure, and reduced toxicity. These findings exemplify the potential of DTX-mPEG-PDLLA micelles for improving the treatment of ovarian cancer with peritoneal metastasis. Furthermore, IP administration of DTX-mPEG-PDLLA may be a potential delivery system for the treatment of other abdominal metastases or primary tumors in the abdominal cavity.

## Data Availability

The original contributions presented in the study are included in the article/Supplementary Material, further inquiries can be directed to the corresponding author.
